# The Depth-Depended Fungal Diversity and Non-depth-Depended Aroma Profiles of Pit Mud for Strong-Flavor Baijiu

**DOI:** 10.3389/fmicb.2021.789845

**Published:** 2022-01-06

**Authors:** Wenchao Cai, Yu’ang Xue, Fengxian Tang, Yurong Wang, Shaoyong Yang, Wenhui Liu, Qiangchuan Hou, Xinquan Yang, Zhuang Guo, Chunhui Shan

**Affiliations:** ^1^School of Food Science, Shihezi University, Shihezi, China; ^2^Hubei Provincial Engineering and Technology Research Center for Food Ingredients, Hubei University of Arts and Science, Xiangyang, China; ^3^Engineering Research Center for Storage and Processing of Xinjiang Characteristic Fruits and Vegetables, Ministry of Education, Shihezi University, Shihezi, China; ^4^Hubei Guxiangyang Baijiu Co., Ltd., Xiangyang, China

**Keywords:** Chinese strong-flavor Baijiu, pit mud, fungal diversity, Illumina MiSeq high-throughput sequencing, electronic nose, aroma

## Abstract

Microorganisms in pit mud are the essential factor determining the style of strong flavor Baijiu. The spatial distribution characteristics of fungal communities and aroma in the pit mud for strong flavor Baijiu from Xinjiang, China, were investigated using Illumina MiSeq high-throughput sequencing and electronic nose technology. A total of 138 fungal genera affiliated with 10 fungal phyla were identified from 27 pit mud samples; of these, *Saccharomycopsis*, *Aspergillus*, and *Apiotrichum* were the core fungal communities, and *Aspergillus* and *Apiotrichum* were the hubs that maintain the structural stability of fungal communities in pit mud. The fungal richness and diversity, as well as aroma of pit mud, showed no significant spatial heterogeneity, but divergences in pit mud at different depths were mainly in pH, total acid, and high abundance fungi. Moisture, NH_4_^+^, and lactate were the main physicochemical factors involved in the maintenance of fungal stability and quality in pit mud, whereas pH had only a weak effect on fungi in pit mud. In addition, the fungal communities of pit mud were not significantly associated with the aroma. The results of this study provide a foundation for exploring the functional microorganisms and dissecting the brewing mechanism of strong flavor Baijiu in Xinjiang, and also contributes to the improvement of pit mud quality by bioaugmentation and controlling environmental physicochemical factors.

## Introduction

Chinese Baijiu is one of the six most renowned distilled liquors in the world, and, according to their flavor characteristics, 12 flavor types, such as strong flavor, sauce flavor, and light flavor, have been formed (Liu M.-K. et al., 2017). Strong-flavor Baijiu (SFB) is a typical representative of Chinese Baijiu, and has a market share of over 70% in China (Zhao et al., 2012; Tao et al., 2014; Liu M.-K. et al., 2017). It is a Baijiu made from grains (sorghum, wheat, corn, rice, etc.) by the traditional solid-state fermentation, distillation, aging, and blending, with ethyl hexanoate as the main body aroma (Tao et al., 2014; Liu M.-K. et al., 2017; Zhang M. et al., 2020). The brewing process of SFB is essentially a process of multiplication and metabolism of various microorganisms, and its biochemical reactions, such as saccharification, alcoholization, esterification, and so on, are all completed in a soil cellar (also known as pit) (Li et al., 2017; Guo et al., 2019). Pit mud (PM) serves as a carrier for diverse and abundant microorganisms in the cellar pits, and its complex microbiota is constantly undergoing natural selection, domestication, enrichment and elimination with the use of cellars, ultimately leading to a stable PM microbial ecosystem with high biological and functional diversity, and the structure and succession of these microbiota influence the aroma and quality of SFB (Wu et al., 2012; Tan et al., 2019; Gao et al., 2020). The microbial species, quantity, community structure, and diversity as well as their interactions directly affect the microecosystem and quality of PM (Liu M.-K. et al., 2017; Guan et al., 2020).

The microbiota of PM that gradually forms after long-term domestication and succession is extremely complex, and a large number of microorganisms in the PM environment cannot be cultured or are difficult to culture. Therefore, traditional isolation and culture technology can only analyze very few culturable microorganisms, making it difficult to objectively reveal the microbial diversity of PM (Cai et al., 2021b). Phosphor lipid fatty acid (PLFA) biomarkers, polymerase chain reaction-denaturing gradient gel electrophoresis (PCR-DGGE), the 16S rRNA gene clone library, single-strand conformation polymorphism (SSCP), fluorescence *in situ* hybridization (FISH) and other modern molecular biology culture-free technologies have overcome the drawbacks of traditional pure culture technology to a certain extent. However, there are still issues such as heavy workloads, high costs, a sensitivity to only the main components in a community and other defects, and these have limitations for the analysis of environmental microbial ecology. Compared with the above methods, high-throughput sequencing technology has obvious advantages for the study of microbial community structure, with low cost, high throughput, extensive coverage, and short time consumption, and it has been shown to be a powerful method for analyzing complex microbiota (Cai et al., 2021a,c). Its combination with bioinformatics analysis can characterize or predict the structure, diversity, and function of microbiota more scientifically and accurately, making the comparison of differences between samples more convincing (Klindworth et al., 2013; Guo et al., 2019).

The microbiota in PM is extremely complex, and its composition is related to cellar age (Tao et al., 2014; Liu M. et al., 2017) and spatial location (Ding et al., 2014; Wang et al., 2014). Tao et al. (2014) found that with increasing cellar age (0–25 years), the microbial community structure of PM was constantly changing and tended to be stable, and the microbial community structure of old PM was in an equilibrium state with high microbial diversity. Liu M. et al. (2017) investigated the internal transcribed spacer (ITS) gene sequence diversity among PM of different cellar ages and showed that *Rhizopus*, *Phoma*, and *Trichosporon* were relatively more abundant in 5-year-old cellar age samples, while *Aspergillus* and *Candida* were most abundant in 100-year-old cellar age samples. Wang et al. (2014) reported that the abundance of *Lactobacillus* decreased with increasing cellar depth, in contrast to *Clostridium*. Ding et al. (2014) detected *Acinetobacter* in the cellar wall but not in the cellar bottom. All these illustrate the abundant microbial diversity of PM. However, current efforts to resolve microbiota at PM in different spatial locations in cellars have focused on bacterial communities, and studies of spatial heterogeneity among fungal communities have rarely been reported.

The purpose of this study is to use high-throughput sequencing technology to comprehensively dissect the fungal community structure in PM at different depths of the special brewing environment of SFB in Xinjiang, meanwhile revealing its correlation with physicochemical factors as well as aroma, which would lay a foundation for directional screening and deep mining of characteristic microbial resources in special brewing habitats for SFB, and provide a theoretical basis for ultimately realizing the modification of microbiota to improve Baijiu quality.

## Materials and Methods

### Sample Collection

In total, 27 PM samples at different depths were collected from 9 cellars with normal fermentation at the same SFB production workshop in Shihezi, Xinjiang, China. For each cellar, PM from the upper layer of cellar wall (10 cm beneath the cellar surface) and middle layer of cellar wall (1.1 m beneath the cellar surface) were collected as upper PM samples and middle PM samples, respectively, while PM from the lower layer of the cellar (cellar bottom) was collected as lower PM samples by a five-point sampling method (Quad corner and centers at the bottom of the cellar). The upper, middle, and lower PM samples were coded as upper1-upper9, middle1-middle9, and lower1-lower9, respectively. The collected samples were sealed in sterile sampling bags and stored at –20°C for further use.

### Determination of Physicochemical Factors

The moisture, pH, humic acid, NH_4_^+^, and nitrogen were determined according to the methods described by Tao et al. (2014), while, the total acid, lactate, and Olsen-P were determined according to the methods described by Wang et al. (2020).

### DNA Extraction

Microbial community metagenomic DNA extraction from PM sample (2 g) was conducted using the QIAGEN DNeasy mericon Food Kit (QIAamp DNA Microbiome Kit, QIAGEN Inc.) in accordance with the manufacturer’s instructions. The DNA extract was checked via 1% agarose gel electrophoresis, and its quantity and quality were measured with a NanoDrop 2000 UV-vis spectrophotometry (Thermo Fisher Scientific Inc., Wilmington, United States) (Cai et al., 2021b). Qualified DNA samples were stored in a –20°C refrigerator for use.

### Polymerase Chain Reaction Amplification and Illumina MiSeq High-Throughput Sequencing

The ITS regions of the fungal rRNA gene were amplified with a forward primer ITS1F (5′-CTTGGTCATTTAGAGGAAGTAA-3′) and reverse primer ITS2R (5′-GCTGCGTTCTTCATCGATGC-3′) (Cai et al., 2021e). The PCR amplification parameters of fungal rRNA gene were set as follows: 95°C for 3 min; 95°C for 30 s, 55°C for 30 s, 72°C for 45 s, 30 cycles; 72°C for 10 min. The PCR reaction mixture consisted of 4 μL 5 × PCR buffer, 2 μL 2.5 mMdNTPs mix, 0.8 μL 5 μmol/L forward primer, 0.8 μL 5 μmol/L reverse primer, 0.4 μL 5 U/μL DNA polymerase, 10 ng DNA template, supplemented to 20 μL with ddH_2_O.

The 30 purified DNA amplicons, diluted to a concentration of 100 nmol/L, were paired-end sequenced on a MiSeq high-throughput sequencing platform in Majorbio Bio-Pharm Technology Co., Ltd. (Shanghai, China).

### Quality Control of the Sequences

The pair-ended sequences generated through MiSeq sequencing were demultiplexed, quality-filtered and merged using the following criteria: (i) the 300 bp reads were truncated at any site receiving an average quality score of <20 over a 50 bp sliding window, and the truncated reads shorter than 50 bp were discarded, reads containing ambiguous characters were also discarded; (ii) only overlapping sequences longer than 10 bp were assembled according to their overlapped sequence. The maximum mismatch ratio of the overlap region is 0.2. Reads that could not be assembled were discarded; (iii) Samples were distinguished according to the barcode and primers, and the sequence direction was adjusted, exact barcode matching, 2 nucleotides mismatches in primer matching.

### Bioinformatics Analysis

The primers and barcode sequences were removed from the high-quality reads via in-house Python scripts from the high-quality reads, meanwhile, all the reads were divided into different samples in the light of their barcodes. Quantitative Insights Into Microbial Ecology (QIIME) package (version 1.9.1) (Caporaso et al., 2010) was applied to perform bioinformatics analysis. The specific operational steps consult a previous report by Cai et al. (2021a). Simply put, UCLUST (Edgar, 2010) was employed to classify high-quality sequences into operational taxonomic units (OTUs) at the threshold of 97% identity, while ChimeraSlayer (Haas et al., 2011) was used to remove potential chimeric sequences from the OTU representative set. Singleton OTUs (OTUs with only one sequence) were removed from all datasets. On the basis of the information extracted from Ribosomal Database Project (RDP, Release 11.5) (Cole et al., 2007) and Sliva (Version 132) (Quast et al., 2013), each OTU was assigned to the lowest taxonomic level with a minimum bootstrap threshold of 80% (Cai et al., 2021d). The OTU table was subsampled correspondingly to adjust the sampling depth for all samples by the “multiple_rarefactions.py program” in the QIIME pipeline. Calculation of alpha and beta diversity was carried out according to the *de novo* taxonomic tree constructed from the representative chimera-checked OTU set by using FastTree (Price et al., 2009). OTU level-based alpha diversity indices, including observed species and the Shannon diversity index, were calculated utilizing the OTU table in QIIME to assess sequence depth and fungal diversity, respectively.

### Aroma Evaluation Using E-Nose

E-nose analysis was performed following the methods described by Cai et al. (2020b) using a Portable Electronic Nose (PEN3, Win Muster Airsense Analytics Inc., Schwerin, Germany).

### Statistical Analysis

Principal co-ordinates analysis (PCoA), permutational multivariate analysis of variance (PERMANOVA), co-occurrence analysis, redundancy analysis (RDA), principal component analysis (PCA), and Procrustes analysis were employed by R software (version 4.0.2).^1^ While linear discriminant analysis effect size (LEfSe) algorithm was applied using Python software (version 3.9.7).^2^

## Results

### Physicochemical Analysis of Pit Mud at Different Depths

**FIGURE 1:**
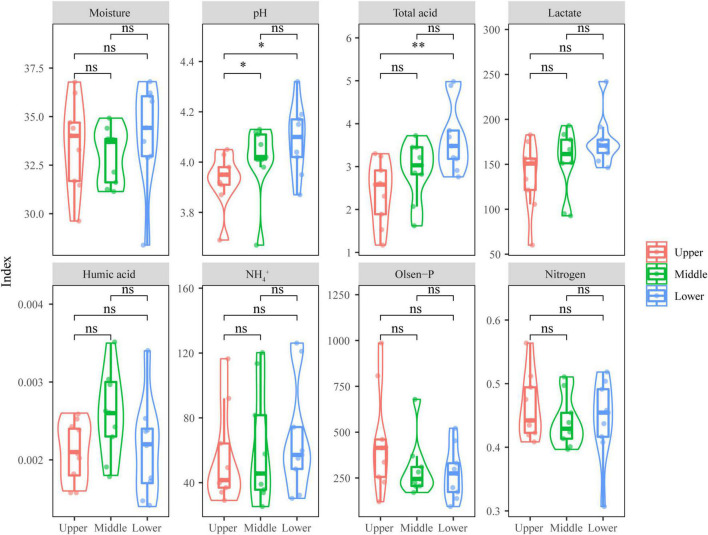
Physicochemical indicators of PM samples at different depths. Significant difference is represented by ^**^ (0.001 ≤ *p* < 0.01), * (0.01 ≤ *p* < 0.05), and ns (*p* ≥ 0.05), respectively.

The physicochemical properties of PM represent the growth environment where PM microbiota resided, and these were important to the growth of PM microbiota. From Figure 1, it can be seen that most of the physicochemical indicators changed insignificantly (*p* > 0.05) with the depth of PM, including moisture, lactate, humic acid, NH_4_^+^, Olsen-P, and nitrogen; only pH and total acid presented a significant difference (*p* < 0.05). The upper PM had the highest Olsen-P content, the middle PM had the highest humic acid content, while the lower PM had the highest moisture, pH, total acid, lactate, NH_4_^+^, and nitrogen contents.

### Fungal α-Diversity Analysis of Pit Mud at Different Depths

Based on the extraction of metagenomic DNA from PM Samples, Illumina MiSeq high-throughput sequencing technology was applied, and a dataset including 1,394,672 high-quality reads of the ITS rRNA gene was generated from 27 PM samples, with an average of 51,655 ± 13,181 (mean ± SD, range from 19,035 to 72,631) ITS rRNA gene reads per sample. At a high threshold identity cutoff level of 97% sequence similarity, 3,394 OTUs were detected. After removing singleton OTUs, the average number of OTUs per sample was 567 ± 223 (range from 165 to 1,024). The specific sequencing information and the number statistics of each taxonomy of PM samples were summarized in Supplementary Table 1.

**FIGURE 2:**
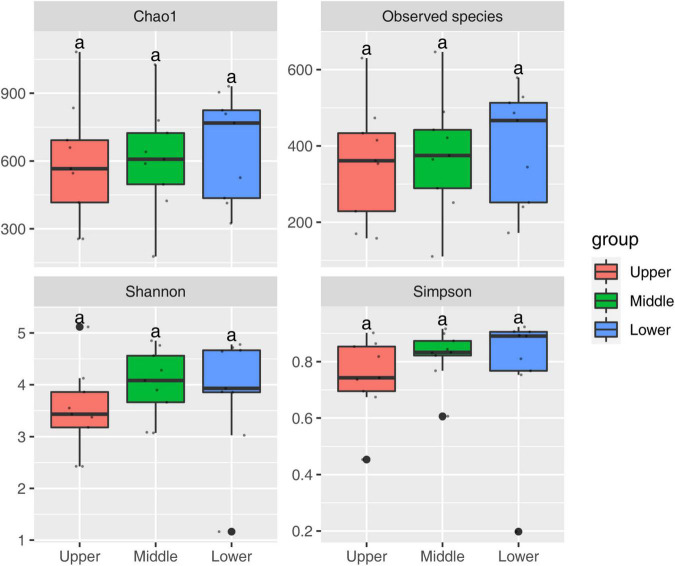
Four α-diversity indexes of PM samples at different depths.

The α-diversity of PM samples at different depths was assessed by Chao1 index and the number of observed species to determine (fungal richness) as well as the Shannon diversity index and the Simpson diversity index to determine (fungal diversity) (Figure 2). Although the difference in these four α-diversity indexes between PM samples of different depths was not significant (*p* > 0.05), it was still observed that all α-diversity indexes ranked from large to small were lower PM > middle PM > upper PM, which illustrated that the lower PM samples had the highest fungal richness and diversity, while the upper PM had the lowest.

### Comparison of Fungal Communities in Pit Mud Samples at Different Depths

**FIGURE 3:**
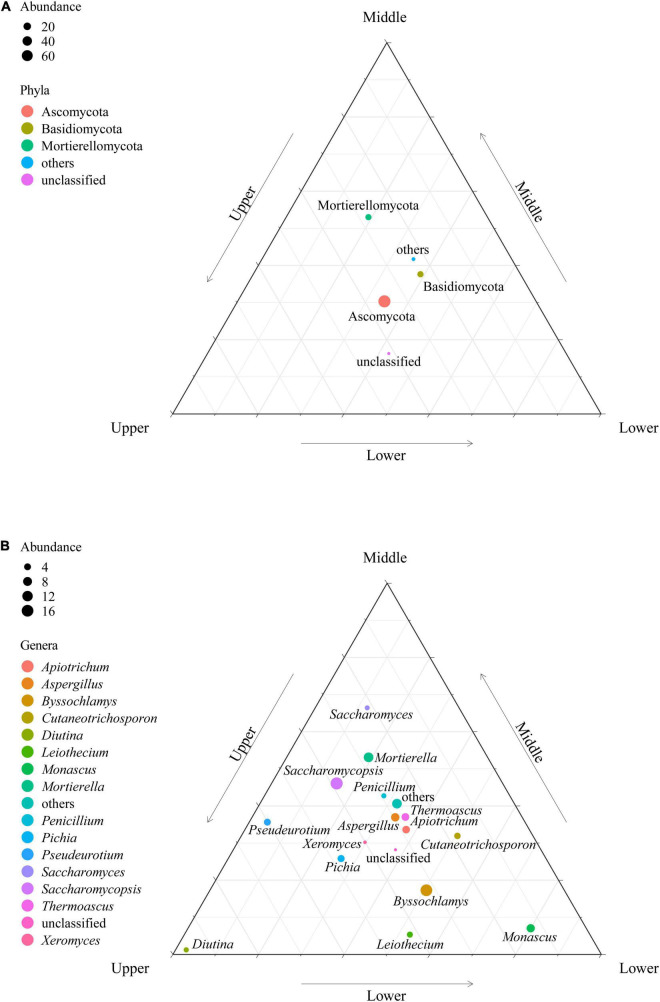
Fungal composition of PM samples at the level of phylum **(A)** and genus **(B)**.

According to the sequence information obtained from high-throughput sequencing, the classifications and relative abundance of fungi in PM at different depths were analyzed at the phyla and genera levels (Figure 3). The fungal phyla/genera in the PM samples were classified as dominant fungal phyla/genera (average relative abundances > 1.00%) and others (average relative abundances < 1.00%) based on relative abundance. A total of 10 fungal phyla were identified in the 27 PM samples. Ascomycota (78.47%), Basidiomycota (10.07%), and Mortierellomycota (9.77%) were dominant fungal phyla in PM, where Ascomycota and Basidiomycota were present in each sample, making them the absolute dominant fungal phyla in PM. At the genus level, 15 out of 138 fungal genera were the dominant fungal genera, namely *Saccharomycopsis* (17.30%), *Byssochlamys* (16.04%), *Mortierella* (9.85%), *Aspergillus* (6.85%), *Monascus* (6.51%), *Thermoascus* (5.45%), *Apiotrichum* (5.21%), *Pichia* (4.35%), *Pseudeurotium* (4.10%), *Cutaneotrichosporon* (3.42%), *Leiothecium* (2.90%), *Diutina* (2.06%), *Penicillium* (1.98%), *Saccharomyces* (1.86%), and *Xeromyces* (1.19%), which accounted for 89.06% of the total sequences. Additionally, *Saccharomycopsis*, *Aspergillus*, and *Apiotrichum* were found in all PM samples, illustrating they were the absolute dominant fungal genera.

**FIGURE 4:**
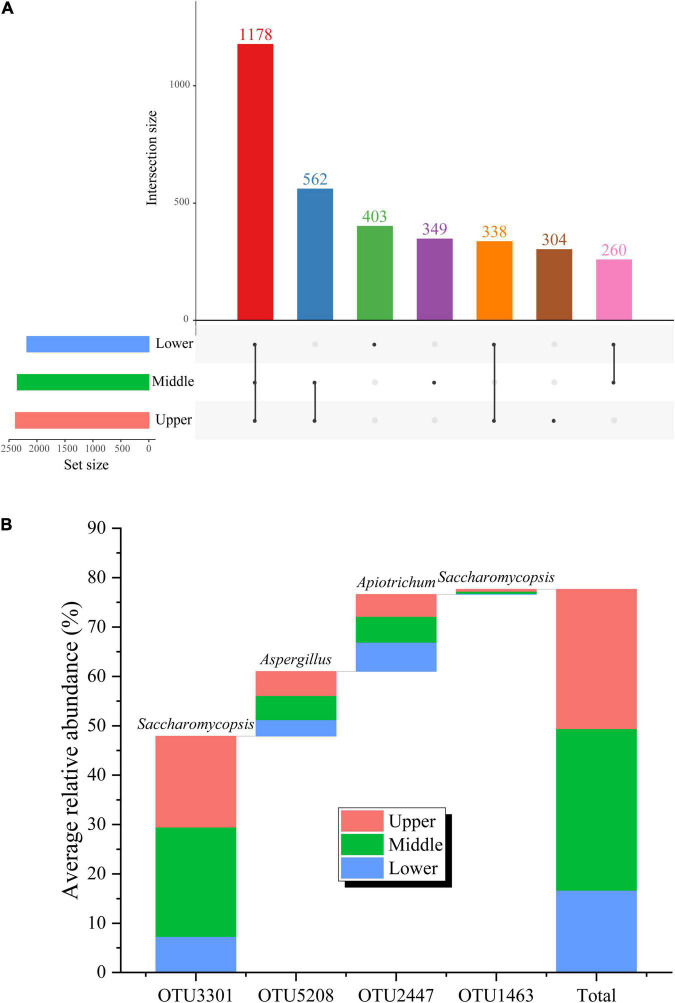
Fungal shared OTUs in PM samples at different depths **(A)** and average relative abundance **(B)** of core OTUs in PM samples.

Further analysis on the fungal communities of PM samples at different depths was performed at the OTU level (Figure 4). A total of 3,394 OTUs were generated in the 27 PM samples included in this study. The upper, middle, and lower PM possessed 304, 349, and 403 unique OTUs, respectively, indicating that the fungal abundance and diversity of the PM also improved with increasing depth, consistent with the results of the α-diversity analysis. Although the PM samples of the different depths all possessed large numbers of unique OTUs, the number of included sequences was only 48,330, accounting for merely 3.47% of all qualified sequences after quality control (Figure 4A). It follows that, despite the fungal community of PM samples at different depths may contain a variety of unique fungal species, their relative abundance is extremely low. Meanwhile, 1,178 OTUs were present in PM at all depths (Figure 4A), among which, OTU3301 (affiliated with *Saccharomycopsis*, 47.89%), OTU5208 (affiliated with *Aspergillus*, 13.12%), and OTU2447 (affiliated with *Apiotrichum*, 13.12%) had relative abundances >1.00% in all 27 PM samples, making them the core OTUs in PM (Figure 4B). The relative abundance of these three core OTUs accumulated up to 76.62%, accounting for 25.95% of all qualified sequences after quality control. The cumulative relative abundance of these three core OTUs was as high as 76.62% (Figure 4B), indicating that OTU3301, OTU5208, and OTU2447, which were affiliated with the three absolute dominant fungal genera (*Saccharomycopsis*, *Aspergillus*, and *Apiotrichum*) in PM, respectively, were core fungal communities in PM.

### Fungal β-Diversity Analysis of Pit Mud at Different Depths

**FIGURE 5:**
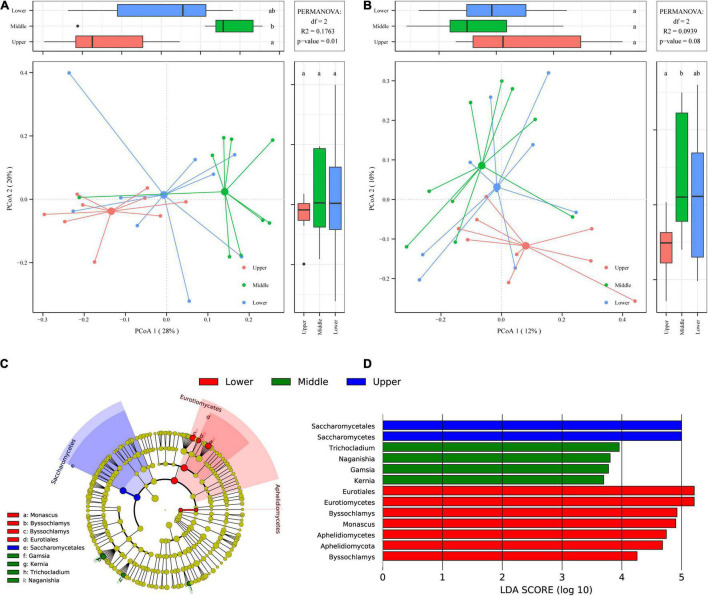
PCoA score plots based on weighted **(A)** and unweighted **(B)** UniFrac distances. Identification of discriminant taxa among PM samples at different depths by LEfSe: Cladogram of the fungal communities **(C).** Horizontal bar chart showing discriminant taxa **(D)**.

To evaluate the variation in fungal communities of PM samples at different depths, PCoA based on weighted (Figure 5A) and unweighted (Figure 5B) UniFrac distances were employed. It is observed in the PCoA plot based on weighted UniFrac distance (Figure 5A) that PM samples at the three different depths of upper, middle, and lower formed three distinct clusters in the horizontal direction from left to right, and the difference between upper PM and lower PM reached a significant level (*p* < 0.05). While in PCoA plot based on unweighted UniFrac distance (Figure 5B), although three clusters were also formed for PM samples at the three different depths, the separation between clusters was not obvious. PERMANOVA, which measures the significance of inter- and intra-group variations (pseudo F-statistic) by permutation of group assignment, further determined the extent of compositional difference among PM samples at the three different depths. It was found that the intergroup variations of PM samples at different depths were extremely significant (*p* = 0.001) in PCoA based on weighted UniFrac distance (Figure 5A), while not significant (*p* > 0.05) in PCoA based on unweighted UniFrac distance (Figure 5B). This reveals that the high abundance of fungi in PM at different depths was extremely different (*p* = 0.001).

LEfSe was carried out with the linear discriminant analysis (LDA) threshold score of 3.00, to find which specific fungal communities caused the highly significant differences (*p* = 0.001) in fungal communities between PM samples at different depths (Figures 5C,D). Thirteen differential fungal communities were significantly enriched (*p* < 0.05) in 27 PM samples, of which 2, 4 and 7 were significantly enriched (*p* < 0.05) in upper, middle and lower PM respectively (Figure 5D).

The significantly enriched (*p* < 0.05) fungal communities in upper PM samples were mainly affiliated to Saccharomycetes. The significantly enriched (*p* < 0.05) fungal communities in middle PM samples were mainly affiliated to four fungal genera, including *Trichocladium*, *Naganishia*, *Gamsia*, and *Kernia*. Whereas the significantly enriched (*p* < 0.05) fungal communities in lower PM samples were mainly affiliated to Eurotiomycetes and Aphelidiomycetes, as well as two fungal genera, *Byssochlamys* and *Monascus*. It’s noteworthy that of all 13 differential fungal communities, four had LDA values ≥ 5: Eurotiomycetes and Saccharomycetes at the class level as well as Eurotiales and Saccharomycetales at the order level, illustrating that they were the fungal communities that contribute most to the highly significant fungal differences (*p* = 0.001) among PM samples at different depths. Meanwhile, Eurotiomycetes (42.77%) and Saccharomycetes (27.86%) were the two fungal classes with the highest average relative abundance in PM (Supplementary Figure 1A), and Eurotiales (42.48%) and Saccharomycetales (27.86%) were the two fungal orders with the highest average relative abundance (Supplementary Figure 1B). It also demonstrated again that the differences between the high abundance fungi in PM were the most significant as revealed by PCoA based on weighted UniFrac distance (Figure 5A).

### Relationships of Physicochemical Factors and Fungal Communities in Pit Mud

**FIGURE 6:**
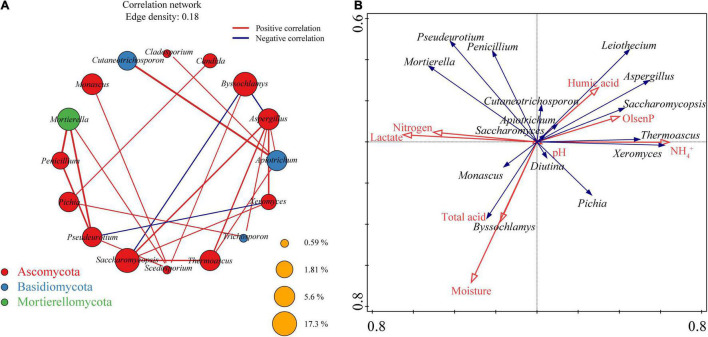
Co-occurrence network depicting the interactions between fungal communities **(A)**. RDA biplot showing the relationship between the dominant fungal genera and physicochemical factors **(B)**.

The environmental conditions in which PM microorganisms live are complex and variable, and these microorganisms, in order to survive and grow, have developed different relationships with each other during domestication to adapt to difficult conditions and to changes in the environment. To reveal these relationships between physicochemical factors and fungi in the microecosystem of SFB fermentation, co-occurrence analysis was performed for all fungal communities identified in PM (Figure 6A), and RDA was performed for the relationships between physicochemical factors and fungal communities (Figure 6B). Sixteen nodes (each representing a fungal genus) and 23 edges (the line between two nodes, with the degree of thickness representing the strength of the correlation) were obtained in the co-occurrence network. All nodes were affiliated to three phyla: Ascomycota (12 nodes), Basidiomycota (three nodes), and Mortierellomycota (one node). The correlation of the co-occurrence network was mainly 16 (69.57%) for intra-phylum microbial correlation and 7 (30.43%) for inter-phylum microbial correlation. Of all the 23 edges, there were 20 co-occurrences relationships, and only three co-exclusive relationships existed, namely, *Byssochlamys* with *Saccharomycopsis*, *Byssochlamys* with *Aspergillus*, and *Pseudeurotium* with *Xeromyces*.

Hubs, also known as high correlation nodes, refer to a class of microorganisms that are associated with a certain number of other microorganisms and are important factors influencing the microbial network in the whole environment (Faust and Raes, 2012). The reduction in the kinds and abundance of hubs will lead to fragmentation of the entire microbial network, resulting in deterioration or even degradation of PM microecological stability (Peura et al., 2015). The hubs (fungal genera with edges ≥ 4) in the co-occurrence network of the fungal communities in the PM were *Aspergillus*, *Apiotrichum*, and *Scedosporium*, which were significantly correlated with six (positively correlated with *Saccharomycopsis*, *Thermoascus*, *Trichosporon*, *Xeromyces*, and *Apiotrichum* and negatively correlated with *Byssochlamys*), four (positively correlated with *Cutaneotrichosporon*, *Aspergillus*, *Thermoascus*, and *Cladosporium*), and four (positively correlated with *Byssochlamys*, *Monascus*, *Pseudeurotium*, and *Mortierella*) fungal genera, respectively. The average relative abundance of all three hubs showed an upward trend with increasing cellar depth, that is, the average relative abundance in upper PM < middle PM < lower PM. Meanwhile, no independent modules were found in the co-occurrence network, and all fungal genera were connected within the same module, suggesting that the hubs maintain the stable operation of the fungal communities in the whole cellar. It is worth mentioning that *Scedosporium* is a non-dominant fungal genus with an average relative abundance < 1.00% in PM, besides, *Candida*, *Cladosporium*, and *Trichosporon* in the co-occurrence network also belong to non-dominant fungal genera.

RDA (Figure 6B) exhibited the possible relationships between fungal communities and physicochemical factors of PM. Except for pH, the seven remaining physicochemical indicators were all found to have large effects on fungal communities in PM, among which three of the most influential physicochemical indicators were as follows: moisture, NH_4_^+^, and lactate. On the basis of these three indicators, all physicochemical indicators can be classified into three clusters: moisture and total acid had basically the same effects on fungal communities in PM, forming the first cluster, humic acid, Olsen-P, and NH_4_^+^ together forming the second cluster, whereas lactate and nitrogen had very similar effects on fungal communities in PM, forming the third clusters. It is worthy of notice that the effect of pH on fungal communities in PM is very weak. Among the 15 dominant fungal genera, *Cutaneotrichosporon*, *Apiotrichum*, *Diutina*, and *Saccharomyces* were less affected by these 8 physicochemical indicators, while the remaining 11 dominant fungal genera received a larger influence. Specifically, moisture and total acid were positively correlated with *Byssochlamys*, *Monascus*, and *Pichia*, humic acid; Olsen-P, and NH_4_^+^ were positively correlated with *Leiothecium*, *Aspergillus*, *Saccharomycopsis*, *Thermoascus*, and *Pichia*; while lactate and nitrogen were positively correlated with *Pseudeurotium*, *Mortierella*, *Penicillium*, and *Byssochlamys*.

### Aroma Analysis of Pit Mud at Different Depths Based on E-Nose

**FIGURE 7:**
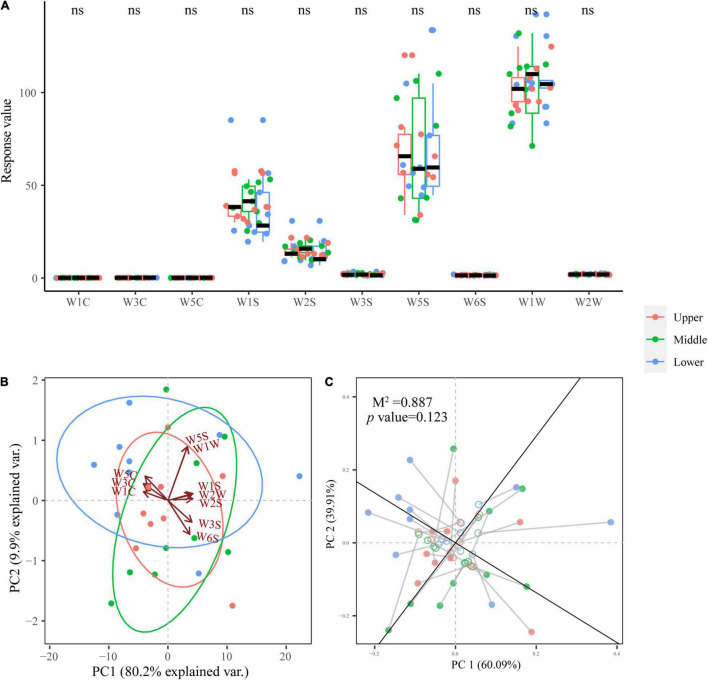
Box plot for aroma profiles of PM samples at different depths **(A)**. PCA biplot based on the aroma profiles of PM samples **(B)**. Procrustes analysis of the correlation between dominant fungal genera and aroma profiles (*M*^2^ = 0.887, *p* = 0.123, 999 permutations) **(C)**.

It is generally believed that PM is related to many crucial aroma compounds in SFB (Gao et al., 2021). Therefore, it is necessary to evaluate the aroma profiles of PM samples with E-nose and to explore the relationship of the dominant fungal genera and aroma. As illustrated in Figure 7A, PM samples at different depths did not show significant differences (*p* > 0.05) in the response values of all 10 E-nose sensors, indicating that their aroma profiles did not differ significantly (*p* > 0.05). The four E-nose sensors with the highest response values ranked in order of magnitude of response value were: W1W, W5S, W1S, and W2S.

PCA (Figure 7B) presents the aroma profiles of PM samples at different depths more intuitively and comprehensively. From the PCA biplot, PM samples at different depths did not show a clear separation or clustering trend, and all three groups of samples overlapped, providing further evidence that PM samples at different depths have similar aroma profiles.

Procrustes analysis based on the PCA of both the dominant fungal genera and aroma of PM samples was employed to reveal the relationship between them, and it was found that the correlation was not significant (*M*^2^ = 0.887, *p* = 0.123 > 0.05) (Figure 7C). This illustrates the fungal communities in PM had no obvious effect on aroma.

## Discussion

PM is one of the main ecological environments inhabited by important functional microorganisms during SFB fermentation. The directed evolution of microorganisms driven by the environmental stress effects of PM as well as the complex material energy metabolism provided the necessary conditions for the formation of various aroma compounds, which had a decisive impact on the characteristic flavor of SFB (Zhao et al., 2012; Wang et al., 2017).

For the first time, high-throughput sequencing was employed to unveil the structure and diversity of the fungal communities in Xinjiang PM at different depths. It was found that the fungal communities in Xinjiang PM, regardless of depth, were classified into three dominant fungal phyla: Ascomycota (78.47%), Basidiomycota (10.07%) and Mortierellomycota (9.77%) (Figure 3). Different from the present findings, Liu M. et al. (2017) used denaturing gradient gel electrophoresis (DGGE) and Illumina MiSeq sequencing to examine the structure of fungal communities in different years of PM from Sichuan Province, China, and identified Ascomycota, Basidiomycota, and Zygomycota as the dominant fungal phyla in Sichuan PM. The discrepancies in the dominant fungal phyla of PM between the two regions may be related to different regional geographic climates and divergences in the soil as well as cellar physicochemical properties.

The fungal richness and diversity of the lower PM were the highest, while that of the upper PM was the lowest (Figure 2). This is in consonance with the observations of Deng et al. (2012) and Ding et al. (2014). It is mainly due to the fact that the upper PM is most susceptible to downwelling of water or cellar opening, with frequent exposure to air and large loss of water, which leads to a rapid decline in moisture and a decrease in microbial species and abundance, resulting in a decline in fungal richness and diversity. The microorganisms in the lower PM would directly exchange with the PM in raw materials (fermenting grains) under the influence of high moisture during SFB fermentation, and enrich a large number of microorganisms, especially brewing microorganisms, thus enhancing the fungal richness and diversity (Deng et al., 2012).

Both PCoA and LEfSe demonstrated highly significant differences (*p* = 0.001) among the high abundance fungi in PM samples at different depths (Figure 5), indicating that the high abundance fungi in PM were more affected by depth. Among these high abundance fungi, Saccharomycetes was significantly enriched (*p* < 0.05) in the upper PM samples, while molds such as *Byssochlamys* and *Monascus* were significantly enriched (*p* < 0.05) in the lower PM samples. This parallels the finding of fungal community structure in PM from Anhui Province, China by Gao et al. (2021). This is because most of the Saccharomycetes are aerobic or facultative aerobic fungi, suitable for growth and reproduction in the upper PM, which has a large contact surface with oxygen and high oxygen content; however, the lower PM is perennial in the soaking of water formed by fermentation and prefers anaerobic, high-pressure, and humid environments, and thus more suitable for molds with stronger environmental adaptability (Zheng et al., 2013; Hu et al., 2016; Liu M. et al., 2017).

The microbiota of any environment or fermentation system is subject to the physicochemical factors of their surroundings, which is also the rationale for continuous domestication, enrichment and elimination of microorganisms in PM. Previous studies have consistently identified the pH of PM as the most influential and broad-ranging physicochemical factor on microbiota and a pivotal environmental stress role in the directed evolution of PM microbiota (Tao et al., 2014; Hu et al., 2016; Zhang M. et al., 2020). The pH of PM would significantly affect the reproduction of microorganisms and their involved biochemical reactions, and an appropriate pH could not only promote alcoholic fermentation, but also facilitate the formation of aroma precursors in SFB to improve product quality (Zhang M. et al., 2020). Interestingly, the present study found a very weak effect of pH on fungal communities in PM (Figure 6B). This may be due to the fact that previous studies on microbiota in PM have mainly focused on bacterial communities, and the range of pH values suitable for bacterial growth is narrow. In contrast, fungal communities typically exhibit a wider range of suitable growth pH values and are therefore less affected by pH (Rousk et al., 2010b; Zhao et al., 2012). However, as the physicochemical indicator that most affected the fungal communities in PM, the levels of moisture in the lower PM (34.13 ± 2.6%) were slightly higher than those in the upper PM (33.57 ± 2.32%) and the middle PM (32.98 ± 1.45%), but the differences were not significant (*p* > 0.05) (Figure 1). During the fermentation of fermenting grains of Baijiu, the raw materials underwent microbial catabolism to produce a large amount of free water, and this moisture with the unused moisture in the fermenting grains continually settled under the action of gravity and formed brownish yellow or brownish viscous liquid (that is yellow water), so that the lower PM had a higher moisture content than the upper PM (Chai et al., 2021; Qian et al., 2021). Meanwhile, various acids were transmitted to the lower PM through the moisture medium, resulting in the highest total acid content and lactate content of the lower PM. This also explains the consistent effects of moisture and total acid on fungal communities in RDA (Figure 6B). Gao et al. (2021) compared the moisture of Anhui PM at different depths and similarly found that the moisture of the lower PM was higher than that of the upper PM; different from the results of this study, however, the moisture of Anhui PM at different depth differed significantly (*p* < 0.05). This may be attributed to the soil environment variability in different regions. Li et al. (2019) measured soil moisture of different depths at 142 sites in Xinjiang and found that moisture increased with soil depth, but the difference in moisture between the 0–50 cm soil layers and the 50–100 cm soil layers was not significant (*p* > 0.05), which was in line with the phenomenon observed in this study at Xinjiang PM.

Moisture and total acid were negatively associated with the majority of dominant fungal genera in PM (Figure 6B), which may be due to the limited availability of free water to PM fungi. Meanwhile, these two physicochemical indicators positively correlated with the abundance of two main molds in the brewing of Baijiu: *Byssochlamys* and *Monascus* (Figure 6B). Molds can produce a large amount of glucoamylase during Baijiu fermentation, which can degrade starch from raw materials into reducing sugar for direct use by yeasts and promote the growth and reproduction of yeasts, thus producing more alcohol (Lv et al., 2012). Among them, *Byssochlamys* can grow under very low oxygen tension, while *Monascus* can secrete acid protease, and adapt to the acidic environment of Baijiu-making (Houbraken et al., 2006; Chen et al., 2011). This could explain the significant enrichment of *Byssochlamys* and *Monascus* in the lower PM with the worst air permeability, the least oxygen contact, and the highest total acid content (Figure 5D). *Byssochlamys* is closely related to cellulose-degrading enzymes (cellulase and glucosidase, etc.) and starch hydrolase (glucose) synthesis (Thorsen et al., 2006; Schuerg et al., 2017; Timo et al., 2017). *Monascus* can secrete glucoamylase, amylase, esterase along with acid protease and has strong saccharification, fermentation as well as esterification ability, which can improve Baijiu yield and quality (Wang et al., 2008; Chen et al., 2011). At the same time, it also produces a variety of secondary metabolites beneficial to the human body, including monasculin, gamma-aminobutyric acid (GABA) and other functional components, which improve the nutritional and health value of Baijiu (Rashmi and Tallapragada, 2015; Wan et al., 2015). Hence, the moisture in PM, as the most critical physicochemical indicator, affects the quality of Baijiu by affecting physicochemical and microbiota.

Generally, the moisture of superior PM is considered to be between 40 and 50%, and its content will directly affect the pH, humus, and microbiota as well as their growth status (Tao et al., 2014; Liu M.-K. et al., 2017; Guo et al., 2019; Liu M.-K. et al., 2019). However, the moisture of Xinjiang PM (range: 28.0–38.7%) (Figure 1) was lower than the normal range (40–50%). This may be explained by the climatic conditions in Xinjiang: Xinjiang Uyghur Autonomous Region is located on the hinterland of Eurasia, far from any ocean, and the overall topographic structure consists of three mountain ranges with two basins: Three mountain ranges (the Altay Mountains, the Tianshan Mountains, and the Kunlun Mountains) surround two huge desert basins (the Junngar Basin and the Tarim Basin) from north to south (Yao et al., 2018). This unique geographic feature greatly affects water vapor transport and spatial distribution, and the three mountain ranges, in particular, block most of the water from the Black Sea, Aral, Caspian, Mediterranean, and Arctic oceans as well as from the south (Du et al., 2021). Geographically, Xinjiang can be divided into three regions, and the Baijiu production area in Xinjiang is mainly clustered in the northern region (which is also the sampling area of this study). The region is located between 42°10′ and 49°10′ latitude as well as 79°59′ and 91°48′ longitude, and the main regional climate is temperate continental arid and semi-arid climate with low precipitation (150–200 mm) and great evaporation potential (1,500–2,300 mm) (Li et al., 2019). These geographical and climatic factors led to the low PM moisture in Xinjiang, which has the least precipitation and the highest evaporation throughout China. Consequently, great attention needs to be paid to the moisture in PM and regulate it in a targeted manner.

Among all eight physicochemical indexes, only pH and total acid presented significant differences (*p* < 0.05) among PM samples at the three depths (Figure 1), indicating that the living environments where the fungal communities of PM at different depths reside differed significantly (*p* < 0.05) only in the contents of acids. Total acid content refers to the total amount of all acidic components in food, including the content of both dissociated acid and undissociated acid. The pH, also known as the hydrogen ion concentration index and acid-base value, reflects the concentration of the acid that has been dissociated, while the PM is slightly alkaline and a small change in hydrogen ion concentration can easily cause pH fluctuation in PM. In this study, the pH of the upper PM was significantly lower (*p* < 0.05) than that of the lower and middle PM, and the pH of the lower PM was the highest (Figure 1). Interestingly, the total acid content of the lower PM was significantly higher (*p* < 0.01) than that of the upper PM samples, indicating that there may be a large amount of undissociated acid in the lower PM. For example, lactate, the major organic acid in PM, is a weak acid, and its Ka in the standard state is 1.37 × 10^–4^, the concentration of which is not linear with the concentration of dissociated hydrogen ions. Other organic acids such as acetic acid, citric acid and pyruvic acid in PM, can also play a certain buffering effect on the whole system, so, the total acid in PM exhibited a paradoxical trend with pH. Moreover, Tao et al. (2014), Liu M.-K. et al. (2017), and Zhang H. et al. (2020) found that hydrogen gas-producing microorganisms (mainly bacteria) such as *Caproiciproducens*, *Syntrophomonas*, and *Methanoculleus* were present in PM and varied in abundance among different depths of PM, so it was speculated that the contradiction between pH and total acid might be related to the different intensity of the metabolism of microbiota to release hydrogen containing gases such as H_2_ in different depths of PM. In addition, H_2_, CO_2_, and acetate produced by gas-producing microorganisms in PM can serve as substrates for *Methanoculleus* (a hydrogenotrophic methanogen) to produce CH_4_ (Wu et al., 2015; Liu M.-K. et al., 2017; Zheng et al., 2020). The release of hydrogen containing gases (H_2_ and CH_4_) under the action of gas producing microorganisms with hydrogenotrophic methanogens, not only decreased the total hydrogen content to raise the pH of PM, but also optimized the anaerobic environment of PM, which was beneficial to the growth and metabolism of lactate degrading bacteria, and then improved the quality of PM. The absence of obvious differences in lactate content among depths may be due to the more mature microbiota and more well-established metabolic functions in PM, and it is speculated that degraded lactate was not the main stress on the metabolism of PM microbiota and the variation range of the microbial genus abundance in PM at each depths varied less, and the lactate content would not have threatened it.

The composition of PM nutrients is a vital marker of PM quality and a vital substrate for microbial growth and reproduction, which, to varying degrees, affects the composition, distribution, and evolution of the PM microbiota. As the three most important nutrients in PM —NH_4_^+^, Olsen-P, and humic acid were positively correlated with *Leiothecium*, *Aspergillus*, *Saccharomycopsis*, *Thermoascus*, *Thermoascus*, and *Pichia* (Figure 6B). NH_4_^+^ is necessary for microorganisms to synthesize various proteins as well as enzymes, and is also a major nitrogen source required for microbial reproduction, growth, and metabolism (Tao et al., 2014; Hu et al., 2016; Zhang M. et al., 2020). A proper amount of NH_4_^+^ is crucial for maintaining PM habitat and improving SFB quality. Olsen-P refers to acid soluble phosphorus and adsorbed phosphorus that can be utilized by microorganisms, they can provide energy to organisms, also constitute a major component of biofilms and nucleic acids, and participate in several biological metabolic pathways such as protein synthesis, esterification of acetic acid with ethanol (Zhang M. et al., 2020). Humic acid is an important organic substance and a major component of humus, which is the main source of carbon, phosphorus, and nitrogen elements in soils and the main nutrients for PM microorganisms (Chen et al., 2020; Zhang M. et al., 2020). NH_4_^+^, Olsen-P, and humic acid provide rapidly utilized nutrients such as nitrogen and phosphorus for microbial growth and reproduction in the environment, which in turn significantly affects the microbial diversity of PM (Tao et al., 2014; Hu et al., 2016). This also explains their positive relationships with *Leiothecium*, *Aspergillus*, *Saccharomycopsis*, *Thermoascus*, *Thermoascus*, and *Pichia*. Although NH_4_^+^, as one of the three physicochemical indicators with the greatest impact on fungal communities in PM (Figure 6B), could be directly utilized by PM microorganisms, the content of which reflected to some extent the ability of PM to supply nitrogen sources required for microbial growth and metabolism, its content is not the higher the better. The different microbiota in PM responds differently to nutrients, thus, a reasonable process approach is required for SFB production to adjust the content of nutrients and balance the structure of microbial communities.

The contents of lactate and nitrogen were negatively correlated or uncorrelated with the absolute dominant fungal genera/core fungal communities (*Saccharomycopsis*, *Aspergillus*, and *Apiotrichum*) or hubs (*Aspergillus* and *Apiotrichum*) (Figure 6B). Lactate, in particular, exhibited high negative correlations with *Saccharomycopsis* and *Aspergillus*, two absolute dominant fungal general/core fungal communities. This resembles the findings of Hu et al. (2016) and Wang et al. (2017). This was mainly due to (i) the massive accumulation of lactate resulting from lactic acid bacteria (LAB) metabolism caused the rise of total acid content in PM, which in turn leads to the reduction or even disappearance of large numbers of microorganisms (mostly adapted to a moderate total acid content) in PM, including dominant fungal genera, core fungal communities, and hubs (Jones et al., 1987; Suetin et al., 2009; Rousk et al., 2010a); (ii) bacteriostatic compounds metabolized by LAB, such as nisin, lactacin and pediocin, could inhibit the growth and reproduction of some microorganisms (Hu et al., 2016; Cai et al., 2019). Although Wang et al. (2014) found that LAB abundance during SFB fermentation decreased with increasing cellar depth, the lactate produced by LAB metabolism will be transferred to the bottom of the cellar with yellow water, such that the lactate content of the lower PM is highest. A moderate amount of lactate could maintain the physicochemical environment of PM, inhibit miscellaneous microbes, acclimate beneficial microbes, and may act as an acid-base regulator in the cellar to maintain the slight acidity of the PM environment and promote saccharification as well as fermentation capacity, but a surge in lactate content would lead to imbalance of PM microbial community, reduced microbiota robustness and even deterioration of cellar mud.

From the results of RDA (Figure 6B), moisture, NH_4_^+^, and lactate were the main driving force for maintaining PM stability and improving its quality. This mainly resulted from: the decline in free water available to microorganisms in PM due to low moisture, the reduction of NH_4_^+^ as a fast-acting nutrient, as well as the increased levels of lactate with bacteriostatic effect on all affected growth and reproduction of PM microorganisms, which then led to imbalance and reduced robustness of PM microbiota, and subsequently affected PM quality (Hu et al., 2016).

Among all the 23 edges in the co-occurrence network, there were 20 pairs of co-occurrence relationships (Figure 6A), indicating that the fungal communities in PM share a stable pattern of symbiosis with each other (Deng et al., 2012). Of these, 12 of the 16 fungal genera were dominant genera, and only four (*Scedosporium*, *Candida*, *Cladosporium*, and *Trichosporon*) were non-dominant genera. This illustrates the complex cooperation and inhibition relationships among fungal communities in PM that lead to the evolution of the structure of fungal communities during long-term reciprocating fermentations, in which dominant fungal genera progressively dominate and achieve dynamic equilibrium, forming high-quality PM (Deng et al., 2012). *Saccharomycopsis*, *Aspergillus*, and *Apiotrichum* were both absolute dominant fungal genera and core fungal communities in PM. They all can degrade macromolecules into carbon sources and small molecule nutrients that can be directly absorbed and utilized by other microorganisms. Meanwhile, *Aspergillus* and *Apiotrichum* were also hubs in co-occurrence networks that maintain the microecological stability of PM and were the most critical fungi in PM.

*Saccharomycopsis* has attracted much attention because of its ability to produce trehalose, amylase, acid protease and β—glucosidase, which are widely used in food, fermentation, biofuel and pharmaceutical industries (Chi et al., 2009; Wang et al., 2011). In Baijiu production, *Saccharomycopsis* is indispensable, it converts starch from raw materials into sugars, which in turn are fermented into ethanol and organic acids. Notably, some glucoamylases produced by *Saccharomycopsis* can digest native starch, which enhances the efficiency of starch degradation in the Baijiu raw materials (i.e., barley, wheat and pea) (Wang et al., 2019).

*Aspergillus* has been detected as the dominant mold in Baijiu production and secretes a wide range of enzymes and metabolites (Hu et al., 2017; Fan et al., 2018; Du et al., 2019; Shoubao et al., 2019; Wang et al., 2019). This genus can secrete various saccharifying hydrolases into its environment, that degrade and convert starch into sugars, to further promote the growth, reproduction and metabolism of bacteria and yeasts (Masayuki et al., 2008; Shoubao et al., 2019). It also produces proteolytic along with other lytic enzymes that contribute to flavonoid formation as well as protein hydrolysis (Masayuki et al., 2008; Gou et al., 2015; He et al., 2019). Meanwhile, *Aspergillus* is considered as the key to the sauce-flavor formation of Baijiu, since it has positive correlations with pyrazines, esters and certain aromatics (Jin et al., 2019).

*Apiotrichum* with an oil-bearing character, which can metabolize a variety of sugars and degrade complex organic contaminants in the environment (Akindumila and Glatz, 1998; Bettencourt et al., 2020; Chen et al., 2021), is a common constituent of forests, meadows as well as gardens and orchards soil and is one of the resident fungi in soils (Vadkertiova et al., 2019). Therefore, it was speculated that it was derived from the soil of Xinjiang, and its function in degrading complex organic contaminants in the environment also partly illustrates its specific contribution as a fungal hub in PM for maintaining microecological stability. *Apiotrichum* has been previously reported only in soy-based foods such as soy sauce (Liu et al., 2021), fermented black soybean curd (Yao et al., 2021), and fermented “hairy” tofu (Benucci et al., 2020). This fungal genus was proved to be positively associated with acids and ketone compounds (Yao et al., 2021), negatively associated with citrulline and arginine (Liu et al., 2021), and related to the biotransformation of tannins and phenols (Spennati et al., 2019). However, it has not been reported for the first time in Baijiu, especially PM, and its specific functions and contributions during fermentation in Baijiu need to be further clarified.

As previously mentioned, the absolute dominant fungal hubs (*Aspergillus* and *Apiotrichum*) in the co-occurrence network were the main factors involved in maintaining the structural stability of the fungal communities in PM (Figure 6A). Among them, *Aspergillus* was positively correlated with NH4^+^, Olsen-P, and humic acid, while *Apiotrichum* did not show an obvious correlation with any physicochemical indicators. To sum up, with clarity on the correlation between fungal co-occurrence network hubs and physicochemical factors, it will help to provide a theoretical basis for further improving the abundance of fungal hubs in Xinjiang PM by directionally adjusting or controlling the physicochemical factors (such as NH_4_^+^, Olsen-P, humic acid) of PM, and then for the stabilization of microbiota in PM.

E-nose combined with PCA (Figures 7A,B) demonstrated that PM at different depths had similar aroma profiles, indicating that the aroma of PM was not affected by depth. And the 4 sensors with the highest response values, in descending order of magnitude: W1W, W5S, W1S, and W2S. W1W is sensitive to terpenes, limonene, pyridine, and sulfur organic compounds that are important for aroma; W5S reacts to nitrogen oxides; W1S is sensitive to methane; while W2S detects alcohols and partially aromatic compounds (Cai et al., 2020a,^2019^). This illustrates that the aroma compounds in PM were mainly terpenes, limonene, pyridine, sulfur organic compounds, nitrogen oxides, methane, alcohols, and aromatic compounds. These aroma compounds have all been widely reported in previous studies of PM, but unfortunately, no significant association between these aroma compounds and the fungal communities in PM was found by Procrustes analysis (Gao et al., 2020, 2021; Zhang M. et al., 2020). This may be affected by the limitations of the E-nose technology itself: E-nose can only identify the profile of the sample aroma but is unable to detect specific aroma compounds qualitatively and quantitatively. In a follow-up study, we will utilize gas chromatography-mass spectrometry technology to further validate this conclusion and explore the impact of microbiota in PM on specific aroma compounds.

## Conclusion

In this study, ITS high-throughput sequencing was used to explore the spatial distribution pattern of fungal communities in PM and explore the relationship between fungal communities and physicochemical factors. The core fungal communities and physicochemical factors were determined, which are important for maintaining the microbial stability of PM. The results also confirmed that the fungal communities of PM had obvious spatial heterogeneity in cellars, and it may have some positive effects on further exploring the specific contributions of PM microbiota at different depths to PM aging and flavor formation. This study enriched the knowledge of the PM microbiota in Xinjiang and also provided certain theoretical support for the maintenance mechanism of cellars and the production of superior PM.

## Data Availability Statement

The datasets presented in this study can be found in online repositories. The names of the repository/repositories and accession number(s) can be found below: https://www.ncbi.nlm.nih.gov/, PRJNA773613.

## Author Contributions

WC: formal analysis, writing—original draft, writing—review and editing, and visualization. Y’aX: investigation, validation, and data curation. FT: project administration. YW: investigation and validation. SY: resources and conceptualization. WL: investigation and data curation. QH: software and validation. XY: resources and supervision. ZG: conceptualization and supervision. CS: project administration and funding acquisition. All authors contributed to the article and approved the submitted version.

## Conflict of Interest

SY and WL were employed by company Hubei Guxiangyang Baijiu Co., Ltd. The remaining authors declare that the research was conducted in the absence of any commercial or financial relationships that could be construed as a potential conflict of interest.

## Publisher’s Note

All claims expressed in this article are solely those of the authors and do not necessarily represent those of their affiliated organizations, or those of the publisher, the editors and the reviewers. Any product that may be evaluated in this article, or claim that may be made by its manufacturer, is not guaranteed or endorsed by the publisher.

## Footnotes

1
https://www.r-project.org/

2
https://www.python.org/

## Supplementary Material

Supplementary Figure 1 Fungal composition of PM samples at the level of class **(A)** and order **(B)**.
