# Supplementary figures and images for: The Depth-Depended Fungal Diversity and Non-depth-Depended Aroma Profiles of Pit Mud for Strong-Flavor Baijiu
Source: Front Microbiol. 2022 Jan 6;12:789845. doi: 10.3389/fmicb.2021.789845 (PMC8770870; doi:10.3389/fmicb.2021.789845)

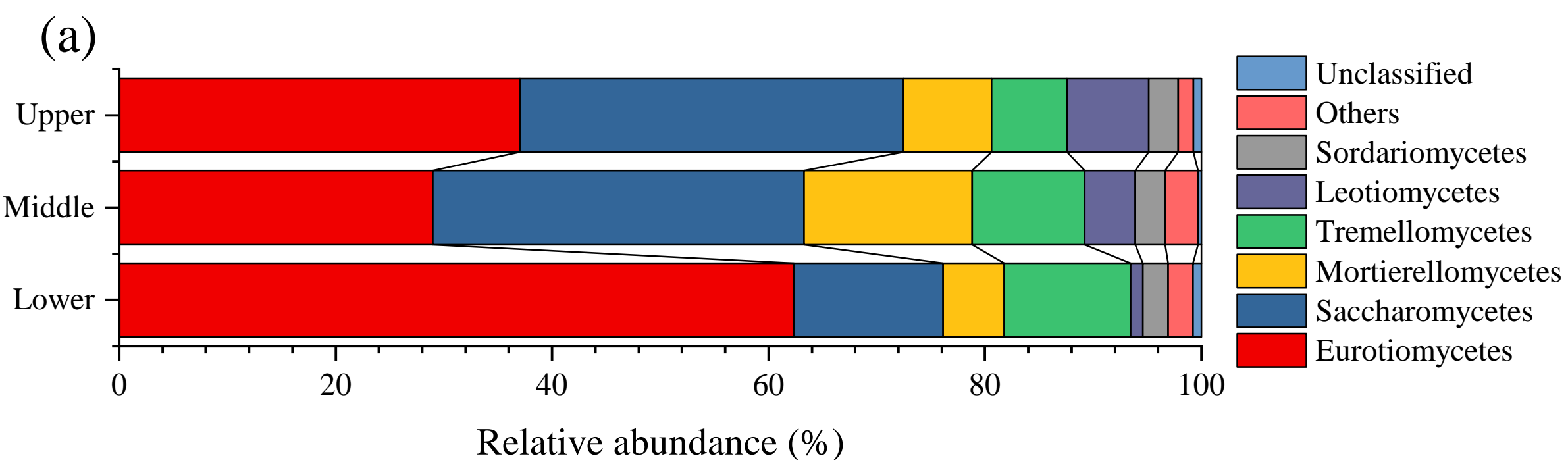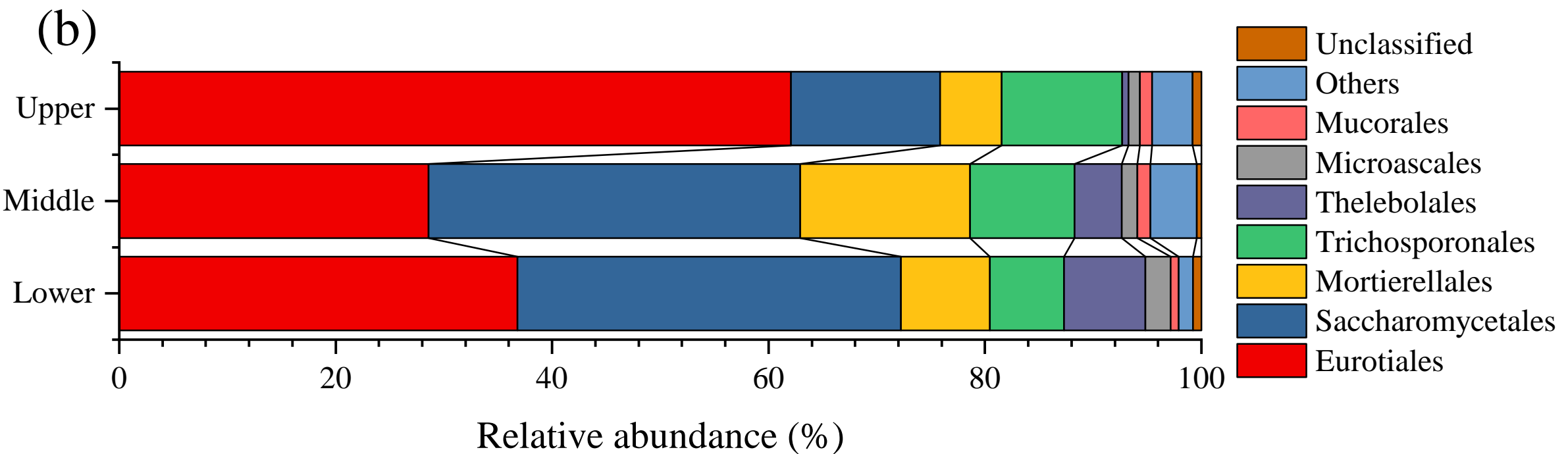

Supplement: Supplementary Figure 1 — Fungal composition of PM samples at the level of class (A) and order (B). [file Data_Sheet_1.PDF]
